# A Full Suite of Histone and Histone Modifying Genes Are Transcribed in the Dinoflagellate *Lingulodinium*

**DOI:** 10.1371/journal.pone.0034340

**Published:** 2012-04-04

**Authors:** Sougata Roy, David Morse

**Affiliations:** Institut de Recherche en Biologie Végétale, Département de Sciences Biologiques, Université de Montréal, Montréal, Québec, Canada; University of Nottingham, United Kingdom

## Abstract

**Background:**

Dinoflagellates typically lack histones and nucleosomes are not observed in DNA spreads. However, recent studies have shown the presence of core histone mRNA sequences scattered among different dinoflagellate species. To date, the presence of all components required for manufacturing and modifying nucleosomes in a single dinoflagellate species has not been confirmed.

**Methodology and Results:**

Analysis of a *Lingulodinium* transcriptome obtained by Illumina sequencing of mRNA shows several different copies of each of the four core histones as well as a suite of histone modifying enzymes and histone chaperone proteins. Phylogenetic analysis shows one of each *Lingulodinium* histone copies belongs to the dinoflagellate clade while the second is more divergent and does not share a common ancestor. All histone mRNAs are in low abundance (roughly 25 times lower than higher plants) and transcript levels do not vary over the cell cycle. We also tested *Lingulodinium* extracts for histone proteins using immunoblotting and LC-MS/MS, but were unable to confirm histone expression at the protein level.

**Conclusion:**

We show that all core histone sequences are present in the *Lingulodinium* transcriptome. The conservation of these sequences, even though histone protein accumulation remains below currently detectable levels, strongly suggests dinoflagellates possess histones.

## Introduction

Unlike typical eukaryotes, dinoflagellate chromatin is permanently organized into a cholesteric liquid crystal structure [1], [2], similar to structures observed in bacteria grown under stress conditions [3] or in sperm cell nuclei [4]. In the dinoflagellates, a combination of several factors may contribute to this structure, including a high concentration of divalent cations [5], a low ratio (1∶10) of basic protein to DNA [6], and amounts of DNA that can range from 1.5 pg/cell (half that in a haploid human cell) in *Symbiodinium* [7] to roughly 200 pg/cell in *Lingulodinium* [8]. The unique chromatin structure in dinoflagellates is presumably a derived characteristic since nuclei in *Perkinsus*, a genus thought to be ancestral to the dinoflagellates [9], have a typical eukaryotic appearance [10].

An additional factor that is also likely to contribute to the unique structure of the dinoflagellate chromatin is the apparent lack of histones. This view is supported by biochemical evidence showing that protein extracts after gel electrophoresis lack the typical and distinctive pattern of histones [11], [12] as well as by microscopic observations showing that nucleosomes are not visible in DNA spreads [13], [14]. Instead of histones, dinoflagellates use histone-like proteins (HLPs) [15], [16]. HLPs of different dinoflagellates are similar but not identical [17], and have been shown to bind DNA and can be modified post-translationally [18], [19].

In general, DNA synthesis is coupled to histone protein synthesis for efficient assembly into nucleosomes. In plants and lower eukaryotes such as yeasts and ciliates, replication dependent histone mRNAs rely mainly on transcriptional regulation to affect histone accumulation in the S phase [20], [21], [22]. The N-terminal region of the histone proteins generally contains a nuclear localization signal (NLS) [23], [24] that binds to the nuclear import family of karyopherins with the help of Nucleosome Assembly Protein (NAP) [23], [24], [25]. Once inside the nucleus, the histones and DNA are assembled into nucleosomes by the help of NAP and other histone chaperone proteins [26], [27]. Certain residues in histone N-terminal end undergo specific post-translational modifications such as acetylation, methylation, phosphorylation, ADP-ribosylation, ubiquitination, sumoylation and biotinylation [28]. Histone modification causes chromatin to reorganize and can result in epigenetic regulation of gene expression as well as affecting other DNA processes such as recombination, repair and replication [29].

A parsimonious explanation for the lack of nucleosomes and histones in dinoflagellate chromatin is that these organisms no longer contain or express histone genes. However, reports of histones H3 and H2A.X mRNA sequences in *Pyrocystis* and *Alexandrium* [30], [31] as well as by retrieval of all core histones and transcripts for two histone-modifying enzymes and a NAP from an environmental sample of dinoflagellates [32] cast considerable doubt on this idea. The environmental sample contains only dinoflagellate sequences, as their amplification exploited a splice leader (SL) sequence specifically trans-spliced to the 5′ end of all nuclear encoded dinoflagellate mRNAs [33]. However, this study could not determine if any one species of dinoflagellate contained the complete set of histones or if the core histones were scattered among many different species and thus unlikely to be functional.

We undertook the present study because a transcriptome profile from the dinoflagellate *Lingulodinium polyedrum* has allowed an in depth analysis of histone and histone modifying genes in a single species. We report here that this species expresses a full set of core histone genes as well as a variety of histone modifying enzymes and histone chaperone proteins at the RNA level. Despite the fact we have not been able to detect histone proteins in *Lingulodinium* extracts the presence and highly conserved sequence of these genes indicates that, in contrast to what has been previously thought, dinoflagellates do indeed have histones.

## Materials and Methods

### Cell Culture

*Lingulodinium polyedrum* cultures (formerly *Gonyaulax polyedra*; strain CCMP1936) were obtained from the Provasoli-Guillard Culture Center for Marine Phytoplankton (Boothbay Harbor, Maine) and grown in a modified seawater medium (f/2) [34]at constant temperature (19±1°C) in 12-h light/12-h dark cycles using cool white fluorescent light at an intensity of 50 µmol photons m^−2^·s^−1^. The beginning of light period is defined as LD 0, and the beginning of the dark period as LD 12. Cultures were grown to a cell density of 12–14,000 cells/mL. The samples were collected from the middle of the dark phase (LD 18) by filtering on Whatman 541 paper supported by a Buchner funnel, and either used immediately or frozen in liquid nitrogen and stored at −80°C until further use.

### Acid Extraction of proteins

Histone proteins were obtained by trichloroacetic acid (TCA) precipitation of the acid soluble protein fraction as described previously [35], [36] with some minor modifications. After washing with 10 volumes of fresh f/2 medium the cells were suspended in ice-cold acid extraction buffer containing 10 mM HEPES pH 7.9, 1.5 mM MgCl_2_, 10 mM KCl, 0.5 mM DTT and 1.5 mM Phenyl methyl sulfonyl fluoride supplemented with 1 X EDTA-free Protease Inhibitor (from Roche) and HCl at a final concentration of 0.25 M. Cells were broken by three one-minute treatments in a bead beater with Zirconium beads at 4°C. The lysate was then incubated on a rotator for 1 hour at 4°C. Insoluble cell debris was removed by two sequential centrifugations at 11,000× g for 10 and 5 minutes, each at 4°C, and the supernatant retained. To this acid soluble fraction, 100% TCA was added drop by drop with simultaneous mixing by inverting the tubes several times until a final concentration of 33% (v/v) TCA was reached. The solution was then incubated overnight at 4°C and the acid soluble proteins were obtained by centrifugation at 16,000× g for 10 minutes at 4°C. To remove the acid, the pellet was carefully washed three times with ice-cold acetone using centrifugation at 16,000× g for 5 minutes at 4°C after each wash. The final pellet was air dried and dissolved in appropriate amount of ddH2O.

As a positive control, *Saccharomyces cerevisiae* (budding yeast) was cultured in 100 ml of 2X YPAD medium at 30°C to mid-log phase (A_260_ = 0.6). Cells were then harvested by centrifugation at 4°C for 5 min at 2,000× g and washed once with 10 volumes of ice-cold sterile Phosphate buffered saline (pH 7.2). All the procedures after this were the same as described above for *Lingulodinium* cells. All protein concentrations were measured using the Bradford assay (Bio-Rad).

### SDS-PAGE and Immunoblotting

*Lingulodinium* and *Saccharomyces* acid soluble proteins along with molecular weight markers (Low Range-BIORAD) were resolved by SDS-15% Polyacrylamide gel electrophoresis (PAGE) as previously described [36]. To compare the protein profiles after electrophoresis, some gels were stained with Coomassie Blue, while others were used for western blotting. Western blotting was performed using commercial rabbit polyclonal antibodies for histones H3 (ab 1791, Abcam, USA) and H2B (sc-10808, Santa Cruz Biotechnology, USA). For Immunoblotting, the proteins from gels were transferred to the Hybond-P PVDF membranes (Amersham Biosciences) using the Transblot SD Semi-Dry Electrophoretic transfer cell (Bio-Rad) following the manufacturer's protocol. After blocking the membranes with 5% Non-fat dry milk in Tris-buffered saline buffer supplemented with 0.05% Tween-20, immuno-reaction was performed with H3 (1∶5000) and H2B (1∶1000) antibodies in the same buffer. After secondary antibody reaction and subsequent washings, the blots were developed with Chemiluminescent substrate (Millipore) and were exposed to the ImageQuant LAS 4000 (GE Healthcare) to capture the chemiluminescence.

In order to test the commercial H3 antibody for cross-reaction with the *Lingulodinium* protein, a tagged version of our H3 was expressed in bacteria. The H3 sequence was cloned by PCR using primers based on the transcriptome sequence (forward primer 5′-CATTACGCCTGACGCTGTCTACGTGC-3′ and reverse primer 5′- GTTAGCGTCTGCTGCTGACGGCTTC-3′) from a 1^st^ strand cDNA sample prepared from Trizol (Invitrogen) extracted RNA using a reverse transcription reaction catalyzed by MMLV RT (Clontech) and the 5′ CDS primer A of the SMARTer RACE cDNA Amplification kit (Clontech). A second PCR, performed on the first PCR product using the forward primer 5′-TCAGTCggatccATGGCCCGCACGAAGCAG-3′ (containing a BamH1 site indicated by small letters) was used to allow directional cloning into the BamH1 and Sma1 restriction sites of the bacterial expression vector pQE30 (Qiagen). The cloned H3 was sequenced to confirm the correct reading frame and used to transform electrocompetent XL1 blue host cells. A single colony grown on LB-agar containing tetracycline and ampicillin was inoculated into 5 mL of the same medium and left to grow overnight at 37°C. One mL of the overnight culture was used to inoculate Twenty ml of fresh prewarmed (37°C) LB medium with antibiotics was inoculated with one mL overnight culture and grown with vigorous shaking at 37°C until OD^600^ of 0.5. H3 expression was induced by adding IPTG to a final concentration of 1 mM and the culture was grown for another 4 hours with shaking at 37°C. One ml of this culture was centrifuged at 5000× g for 3 minutes at 4°C and the cell pellet resuspended directly in 50 µl SDS-PAGE sample buffer and heated at 95°C for 5 min. The samples were centrifuged to remove debris and 30 µl of sample was loaded onto a 15% polyacrylamide gel. XL1 blue cells containing an empty vector were used as a control. Electrophoresis, transfer and immunoblotting were carried out as above.

### Mass Spectrometric analysis

The total acid soluble protein pellet in acetone was also used for mass spectrometric analysis. Also, after fractionating the yeast and *Lingulodinium* acid soluble proteins in SDS 15% PAGE, the gels were stained with Coomassie Blue and several regions were excised from the gel, both from the *Lingulodinium* and yeast samples. The excised bands were destained and sent to the proteomic facility of l'Institut de recherche en immunologie et en cancérologie (IRIC) in Montreal, Canada. The tryptic digestion and LC-MS/MS sequencing for both the total acid extracted proteins and fractionated gel-excised bands were performed at the IRIC.

### Bioinformatic and Phylogenetic Analysis

The sequences for histones and histone modifying enzymes reported here were retrieved from a *Lingulodinium* transcriptome assembled from roughly 300 million 76 bp Illumina paired end reads combined from several times under a LD cycle and conditions (manuscript in preparation, GenBank Accession numbers JO692619 through JO767447). The Illumina sequencing and assembly was performed at the Genome Quebec sequencing facility. The number of reads corresponding to each histone sequence was determined for RNA samples prepared over LD 6 and LD 18 cell cultures and reported as number of histone reads present per million. The number of reads for the histone sequences in the wild potato *Solanum chacoense* was retrieved from a similar project undertaken concurrently with the *Lingulodinium* samples.

Phylogenetic analysis was performed using an online tool obtained from the website www.phylogeny.fr [37]. Our workflow used the software MUSCLE to align the histone sequences, curation by GBlocks, phyML bootstrapping (100 times) to construct the tree and TreeDyn to visualize the tree. The same workflow was followed for all the phylogenetic analysis.

## Results

### All core histone and many histone modifying enzyme sequences are present in the *Lingulodinium* transcriptome

Analysis of a recent Illumina sequencing run (manuscript in preparation) identified the entire set of core histones, namely H2A, H2B, H3 and H4 from the dinoflagellate *Lingulodinium polyedrum* (Table 1). Partial splice leader sequence [33] was recovered from at least one of the histone sequences and all the sequences are GC-rich, a common characteristic of the dinoflagellate sequences [31]. In addition, *L. polyedrum* also expresses genes encoding enzymes that post-translationally modify histones, such as histone lysine methyltransferase (KMT), histone arginine methyltransferase (PRMT), histone acetyltransferase (KAT) and also histone deacetylases (from both HDAC and sirtuin 2 superfamilies) (Table 2). We also found histone chaperone proteins, which assists in nucleosome formation and chromatin remodelling (Table 2). *Lingulodinium* thus expresses a wide range of genes responsible for making and modifying nucleosomes.

**Table 1:** Description of histone sequences and their relative abundance in *Lingulodinium*.

Histone	*S. chacoense*	*L. polyedra*			
		Sequence ID	GC content	LD 6	LD 18
	(reads/million)			(reads/million)	(reads/million)
H2A	67	JO760634	64%	4	4
		JO759158	69%	2	2
		JO731189	55%	6	6
H2B	30	JO694219	65%	2	2
		JO720817	68%	1	2
H3	124	JO722862	66%	2	3
		JO740554	75%	1	1
		JO753891	65%	2	2
H4	63	JO717937	70%	2	2
		JO719134	66%	3	3

**Table 2:** Description of histone modifying enzymes and histone chaperones.

Protein ID	Hit protein family	Hit Accession Number	E-Value	Similarity	GC content
JO734372	KAT, ELP3	XP_002773536.1	1 e^−71^	67%	67.9%
JO732038	KAT, ELP3	XP_002773536.1	6 e^−72^	78%	65.9%
JO710977	HDAC	XP_001758783.1	3 e^−70^	60%	66.9%
JO734243	HDAC	BAB10370.1	9 e^−45^	67%	66.7%
JO742233	HDAC	XP_001625421.1	1 e^−71^	68%	72.3%
JO743978	HDAC	XP_002514660.1	1 e^−71^	67%	68.5%
JO724091	HDAC, SIR2	XP_003057268.1	2 e^−82^	67%	67.2%
JO726045	HDAC, SIR2	XP_002508530.1	1 e^−76^	70%	73%
JO733933	HDAC, SIR2	XP_003057268.1	4 e^−75^	67%	69.3%
JO726372	KMT, SET	XP_003195141.1	2 e^−30^	51%	68.4%
JO694016	KMT, SET	XP_002785418.1	4 e^−17^	49%	73.5%
JO752203	PRMT	NP_001150868.1	5 e^−64^	56%	69.3%
JO723144	PRMT	NP_001003645.1	6 e^−49^	60%	65.6%
JO735881	PRMT	XP_001945590.2	8 e^−62^	62%	69.4%
JO747341	NAP	XP_002764795.1	2 e^−32^	55%	64.3%
JO745850	NAP	XP_002764795.1	6 e^−34^	50%	70.1%
JO738268	NAP	XP_002764795.1	2 e^−26^	54%	61.7%
JO761496	NAP	XP_002764795.1	2 e^−38^	57%	65.7%
JO748499	ASF1-like	XP_758562.1	1 e^−19^	57%	69.3%
JO750428	NAP-C	ADE76527.1	6 e^−63^	49%	69.3%

### Phylogenetic grouping identifies at least two major variants of all histone sequences within *Lingulodinium*

**Figure 1:**
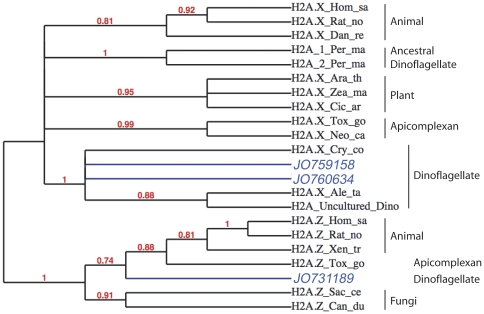
Two variants of Histone H2A in *Lingulodinium*. The cladogram of histone 2A.X and Z variants shows representatives from mammals, plants, fungus and members of the superphylum Alveolata. The representative sequences were obtained from Pubmed database and bear the first three letters from genus followed by two letters from species. The values in red at each node indicate the respective Bootstrap support value. *Lingulodinium* sequences are coloured in blue.

Surprisingly, the *Lingulodinium* transcriptome contains at least two variants of each histone sequence. We thus performed phylogenetic analyses to provide insight into the relationship between the different histone variants. Among the three H2A sequences retrieved, two belong to class H2A.X while the other groups with the eukaryotic H2A.Z proteins (Figure 1). This is the first report of a Z – like variant of histone H2A in any dinoflagellate. The two H2A.X sequences, JO760634 and JO759158, both contain a signature SQEF motif at the C-terminal end that is common to all dinoflagellate H2A.X sequences known so far [32] and as expected, all the dinoflagellate H2A.X variants cluster together. Interestingly, the two *L. polyedrum* H2B proteins belong to two different clades, one common to other dinoflagellate H2B (JO720817) and the other (JO694219) grouping within the superphylum Alveolata along with the ciliates and apicomplexans (Figure S1). Similarly, there are two well supported clades of H3 sequences, one phylogenetically indistinguishable from other eukaryotic H3 sequences and the other divergent (JO753891) form also found in *Pyrocystis lunula* (Figure S2). Unfortunately, there is insufficient phylogenetic resolution to determine the origin of the *Lingulodinium* H4 proteins (Figure S3). In general, however, it seems *Lingulodinium* contains not only a dinoflagellate specific histone but also an additional sequence with a more divergent origin.

### Histone mRNAs abundance levels are uniform throughout

Replication –dependent histone sequences tend to accumulate during the S-phase of the cell cycle. In *Lingulodinium*, S-phase begins in the middle of the dark phase (LD18) for cells grown under a 12∶12 L∶D cycle [38]. We therefore compared the number of sequence reads in a sample from mid-day (LD6) with the LD18 sample. No significant variation in the mRNA abundance between the day and night is supported by the data (Table 1). In general, all the histone mRNAs seem to be of low abundance. By way of comparison, we found *Lingulodinium* histone mRNA abundance to be roughly 5 to 25-fold lower than in the plant *Solanum chacoense*.

### Histone protein accumulation is below current detection limits

**Figure 2:**
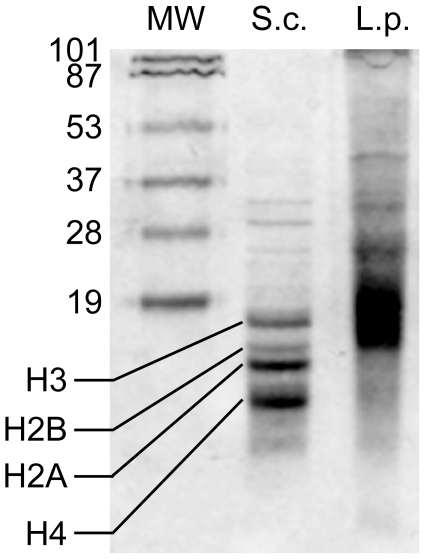
The acid soluble protein profiles of *Lingulodinium* and Yeast differ. A Coomassie blue stained gel containing roughly equivalent amount of acid extracted proteins from *Lingulodinium* and yeast in SDS-15% PAGE is shown here. The regions of the gel corresponding to yeast histones were excised and analysed by LC-MS/MS.

To reconcile the apparent lack of nucleosomes in dinoflagellates with the expression of all core histone transcripts in *Lingulodinium*, we evaluated the extent of histone protein accumulation using more sensitive techniques than those used previously. As shown previously [12], [15] acid extracted proteins from *Lingulodinium* do not have the typical pattern of histones such as found in yeast extracts using SDS PAGE followed by Coomassie blue staining (Figure 2). We used LC-MS/MS to analyze the *Lingulodinium* acid extracted proteins, and both the entire acid extracted protein fraction as well as acid extracted proteins that had been further fractionated by SDS-PAGE into the size range of yeast histones were tested. None of the histone core sequences from *Lingulodinium* were found in any of our samples although we were able to detect *Lingulodinium* histone-like protein, as expected (Table 3, Table S1). As a control, the same experiment was performed with an acid extracted fraction of a yeast extract, and histone sequences H2A and H2B were readily detected (Table 3).

**Table 3:** Proteins found by LC-MS/MS sequencing of total acid soluble proteins from *Lingulodinium* and yeast.

	Type of Protein	No. Proteins (≥2 peptides)	Confidence	Species Hit
*L. polyedrum*	Histone like protein	1	9 e^−26^	*L. polyedrum*
	Perilipin-4	3	7 e^−23^	*Bos taurus*
	Photosystem II 12 kDa extrinsic protein	3	3 e^−37^; 4 e^−36^; 2 e^−35^	*Heterocapsa triquetra*
	Kinesin-K39	1	5 e^−13^	*Leishmania mexicana*
	Elongation factor-1á	2	0	*H. triquetra*
	Malate dehydrogenase	1	1 e^−115^	*H. triquetra*
	Peptidoglycan domain containing protein	1	5 e^−09^	*Tetrahymena thermophila*
*S. cerevisiae*	H2A-1	1	0	*S. cerevisiae*
	H2B-1	1	0	*S. cerevisiae*

**Figure 3:**
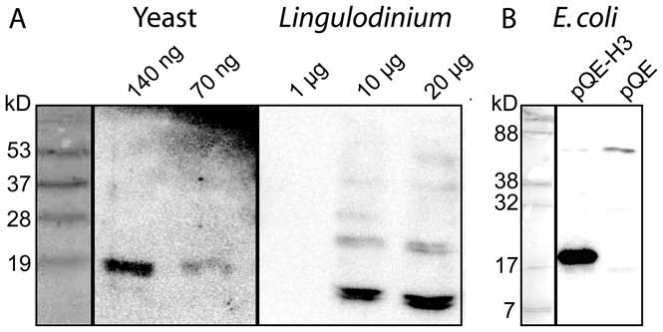
Histone H3 protein levels in *Lingulodinium* are below current immunodetection limits. (A) Acid extracted proteins electrophoresed on SDS-15% PAGE were subjected to Western blot analysis using a commercial H3 antibody. For the yeast and *Lingulodinium* samples, the value above each lane indicates the amount of protein loaded in micrograms, and the samples were run and treated with antibodies concurrently. No signal is detected in the *Lingulodinium* sample at a position corresponding to the yeast H3. (B) Western blots, performed using the same anti-H3 and an H3-expressing *E. coli* strain or an *E. coli* strain containing only the empty vector, demonstrate cross-reaction of the antibody with the *Lingulodinium* H3.

In a separate approach, we tested *Lingulodinium* acid extracted proteins for a cross reaction with histone antibodies. We first tested a commercial anti-H3 directed against an epitope that shared 92% sequence identity with the *Lingulodinium* sequence. This antibody detected the yeast H3 with as little as 0.07 µg of total acid extracted protein, whereas as much as 20 µg of acid extracted protein from *Lingulodinium* did not show a reaction with any protein corresponding in size to the yeast H3 band (Figure 3). The high protein load of *Lingulodinium polyedrum* extracts show cross-reacting proteins with a significantly different mobility from the yeast H3, but the identity of these proteins is unknown. We also tested an antibody raised against the full length H2B sequence of mammalian origin, and again the antibody was unable to detect any band corresponding in size to that of yeast H2B (Figure S4). Again, at high concentrations of protein the antibody showed a cross reaction with a band with reduced mobility (∼30 kD) whose identity is also unknown. As a caveat, however, the H2B used to generate this antibody is only 63% similar to the predicted protein produced by the *Lingulodinium polyedrum* H2B.

## Discussion

Nucleosomes are the basic structural and functional unit of chromatin in most eukaryotes, and are formed when roughly 150 bp of DNA wrap around a histone octamer (two each of H2A, H2B, H3 and H4). Dinoflagellates different from other eukaryotes in that DNA spreads do not show nucleosomes [39], [40], [41], 150 bp DNA fragments of DNA are not protected from microccocal nuclease digestion [14], [42] and gels of basic proteins do not show the typical histone protein pattern [15]. This general rule for dinoflagellates has only two known exceptions, the binucleate dinoflagellates such as *Peridinium balticum* (which have both typical eukaryotic and dinoflagellate nuclei) [43], [44] and members of the endoparasitic *Perkinsus* whose nuclei resemble those in a typical eukaryotic cell [10]. *Perkinsus marinus* is considered to be the ancestor of the dinoflagellate lineage [9], and not only contains all the core histone sequences [45] but lacks the HLPs found in other dinoflagellates.

Recently, high throughput sequencing has revealed that environmental samples of dinoflagellates transcripts contain not only the four core histones, but also two histone modification proteins and a NAP [32]. However, while these sequences are clearly dinoflagellate in origin, based on the distinguishing SL sequence at the 5′ end [33], it is not clear if they are all expressed in the same species. We show here that a single species of dinoflagellate expresses all the core histone (Figures S5, S6, S7, S8) as well as a wide range of histone modifying enzymes and histone chaperone proteins (Table 1 and 2). Furthermore, the gene profile is surprisingly complex, with at least two different variants of predicted histone sequence, one relatively close to other eukaryotic histones and the other more divergent (Figure 1 and Figures S1, S2, S3).

Among the core histones, histone H2A has several subtypes including H2A.1 and 2, H2A.X and H2A.Z. These subtypes each contain signature sequence elements that have been conserved throughout evolution and allow them to be readily identified [46], [47]. In mammals, all the major variants of H2A are present in varying proportions, whereas lower eukaryotes often replace the more common H2A.1 and 2 subfamily with H2A.X [48]. *Lingulodinium* also contains the H2A.X variant and in addition, an H2A.Z-like subtype previously unreported in dinoflagellates (Figure 1). These subtypes are thought to have specific functions, with H2A.X directly involved in DNA repair and genome integrity, which requires the phosphorylation of the C-terminal serine (S) of the SQ(D/E)(M/Y/F) motif [49], and H2A.Z involved in chromosome segregation, cell cycle progression and regulation of expression of cyclin genes, which is mediated by the H2A.Z localized in the promoter regions of these genes [50]. For the H2B and H3 histones, *Lingulodinium* maintains a general eukaryote form in addition to a divergent form common to other dinoflagellates (Figures S1, S2). Interestingly, two of the three H3 sequences in *Lingulodinium* conserve the key post-translational modification sites K4, K9, K27, K36 and K79 [51], while the other divergent forms lack the K27/K36, as in *Pyrocystis* H3 and K79 as in *Karlodinium* H3. For H4, we found two sequences (Figure S3), all with a conserved K20 site, which has been linked to transcription repression upon methylation [51]. Thus, the presence of all core histones, the conservation of sites typically modified, as well as the presence of histone modifying enzymes in the transcriptome (Table 2), all suggest that *Lingulodinium* should accumulate histone proteins.

We had originally anticipated that the amount of histone proteins expected for *Lingulodinium* could be estimated by assuming that the amount of protein produced from a transcript will be proportional to the amount of message independent from the organism in which the transcript is found. We therefore compared the amount of histone transcripts in *Lingulodinium* with those of the plant *Solanum chacoense*, as RNA samples from both were prepared, sequenced and analysed concurrently. In general, the abundance of histone messages in *Lingulodinium* is roughly 30 times less than that in *S. chacoense* (Table 1) and roughly 60-fold less than that reported for yeast [52]. However, immunoblotting was unable to detect H3 in *Lingulodinium*, even when the amount of *Lingulodinium* protein was 300 times greater than yeast. Furthermore, histone proteins were not detected by mass spectrometry (Table 3), either in total or gel fractionated acid soluble extracts, even though other proteins detected in the extracts had similar transcript levels as the *Lingulodinium* histones (Table 4). Thus, it seems histone abundance may be lower than would be predicted. It might also be of interest to test different extraction procedures for histones to see if this aids detection.

**Table 4:** mRNA abundance of expressed proteins detected by LC-MS/MS in an acid-extracted protein fraction.

Accession number	LD 6 reads	LD 18 reads
JO757244	1	1
JO711184	3	3
JO741176	1	1
JO735533	2	2
JO698965	6	5
JO760395	4	3
JO764129	6	5

Histone modification has been linked to several functions such as chromatin remodelling and epigenetic regulation [53], and thus the finding that the *Lingulodinium* transcriptome also contains histone acetyltransferase and deacetylase enzymes as well as methyltransferases (Table 2) supports a role for histones in regulating gene expression. However, it must be noted that while histone deacetylases have a strong link to gene repression and heterochromatin formation [28], [54], [55], they can also target non-histone proteins and regulate DNA binding affinity, protein stability and protein-protein interaction, as well as modulate enzyme activity [56]. Sirtuin family proteins, deacetylases overrepresented in our transcriptome, were also reported in prokaryotes and archeae [57] where they function to regulate metabolism through important enzymes like acetyl-CoA synthetase [58]. Similarly, the SET domain K-methyltransferase that methylates histones can also methylate diverse proteins such as cytochrome *c* and the large subunit of Rubisco [59], [60]. A SET domain histone methyltransferase (NUE) has been reported in the pathogenic bacteria *Chlamydia trachomatis* [61]. Thus, it is possible the histone modifying enzymes in *Lingulodinium* might modify proteins other than the core histones. One prospective substrate could be the *Lingulodinium* HLPs, which have been reported to be acetylated [62]. Similarly, histone chaperone proteins also have important alternative roles other than those related to nucleosome assembly. NAP family proteins specifically interact with B-type cyclin [63], [64] and play a role in regulating cell cycle [65]. It would be of interest to determine if any of the histone modifying enzymes are, unlike the histones themselves, detectable immunologically.

The abundance of histone mRNA in *Lingulodinium* is between 5- and 25-fold lower than in the higher plant *Solanum chacoense* depending on the histone (Table 1). In eukaryotes, histones are found in both replication-dependent and replication-independent classes [66], with the mRNA abundance of replication-dependent histones coupled to the cell cycle as expected [67]. Transcriptional and posttranscriptional regulation can result in a 15- to 30-fold increase in mRNA accumulation with a peak during mid S phase [68], [69]. A comparison of histone mRNA levels at LD 6 and LD 18 (Table 1) does not show preferential abundance during the LD 18, the peak of S-phase in *Lingulodinium* [38], [70]. Thus, histone transcript accumulation is independent from the cell cycle in *Lingulodinium*.

Our results with *Lingulodinium* show that all core histone transcripts are present in a single species. Although histone protein levels remain below our current limit of detection, the presence of all four core histone proteins, the conservation of their sequence, and the presence of a large number of histone modifying enzymes all support the hypothesis that dinoflagellates have histones.

## Supporting Information

Figure S1 **Cladogram of histone H2B.** The cladogram of histone sequences shows representatives from mammals, plants, fungus and members of the superphylum Alveolata. The representative sequences were obtained from Pubmed database and bear the first three letters from genus followed by two letters from species. The values in red at each node indicate the respective Bootstrap support value. *Lingulodinium* sequences are coloured in blue.
(JPG)

Figure S2 **Cladogram of histone H3.** The cladogram of histone sequences shows representatives from mammals, plants, fungus and members of the superphylum Alveolata. The representative sequences were obtained from Pubmed database and bear the first three letters from genus followed by two letters from species. The values in red at each node indicate the respective Bootstrap support value. *Lingulodinium* sequences are coloured in blue.
(JPG)

Figure S3 **Cladogram of histone H4.** The cladogram of histone sequences shows representatives from mammals, plants, fungus and members of the superphylum Alveolata. The representative sequences were obtained from Pubmed database and bear the first three letters from genus followed by two letters from species. The values in red at each node indicate the respective Bootstrap support value. *Lingulodinium* sequences are coloured in blue.
(JPG)

Figure S4 **Histone H2B protein is not detected in** ***Lingulodinium*** **(TIFF).** Western blotting with H2B antibody is shown here. The amount of protein (in micrograms) per lane is written above each lane.
(PDF)

Figure S5 **Alignment of H2A sequences.** Multiple sequence alignment of histone H2A from yeast, human and *Lingulodinium* is shown.
(JPG)

Figure S6 **Alignment of H2B sequences.** Multiple sequence alignment of histone H2B from yeast, human and *Lingulodinium* is shown.
(JPG)

Figure S7 **Alignment of H3 sequences.** Multiple sequence alignment of histone H3 from yeast, human and *Lingulodinium* is shown.
(JPG)

Figure S8 **Alignment of H4 sequences.** Multiple sequence alignment of histone H4 from yeast, human and *Lingulodinium* is shown.
(JPG)

Table S1 **LC-MS/MS identification of acid soluble proteins from** ***Lingulodinium*** **extracts fractionated on SDS PAGE.**
(DOCX)
