# Supplementary figures and images for: A Full Suite of Histone and Histone Modifying Genes Are Transcribed in the Dinoflagellate Lingulodinium
Source: PLoS One. 2012 Apr 4;7(4):e34340. doi: 10.1371/journal.pone.0034340 (PMC3319573; doi:10.1371/journal.pone.0034340)

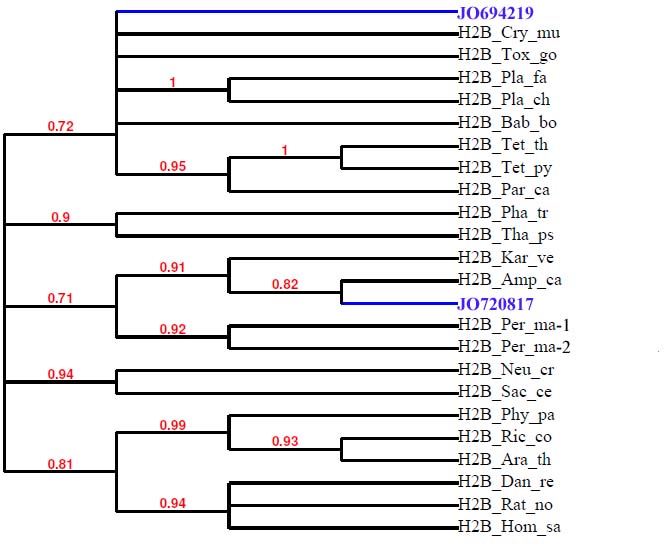

Supplement: Figure S1 — Cladogram of histone H2B. The cladogram of histone sequences shows representatives from mammals, plants, fungus and members of the superphylum Alveolata. The representative sequences were obtained from Pubmed database and bear the first three letters from genus followed by two letters from species. The values in red at each node indicate the respective Bootstrap support value. Lingulodinium sequences are coloured in blue. (JPG) [file pone.0034340.s001.jpg]

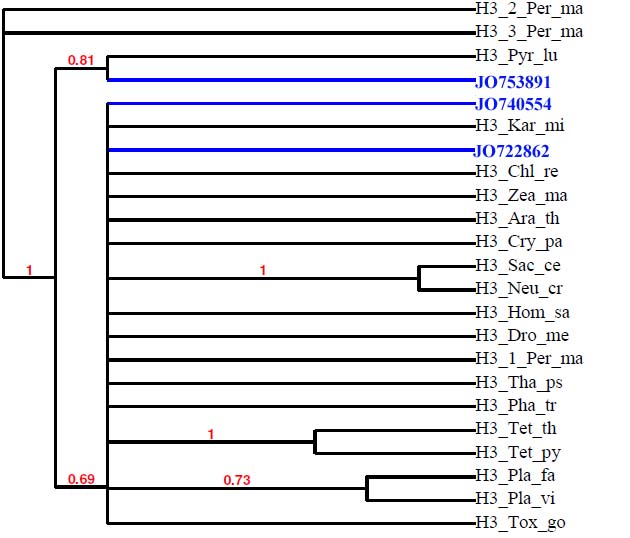

Supplement: Figure S2 — Cladogram of histone H3. The cladogram of histone sequences shows representatives from mammals, plants, fungus and members of the superphylum Alveolata. The representative sequences were obtained from Pubmed database and bear the first three letters from genus followed by two letters from species. The values in red at each node indicate the respective Bootstrap support value. Lingulodinium sequences are coloured in blue. (JPG) [file pone.0034340.s002.jpg]

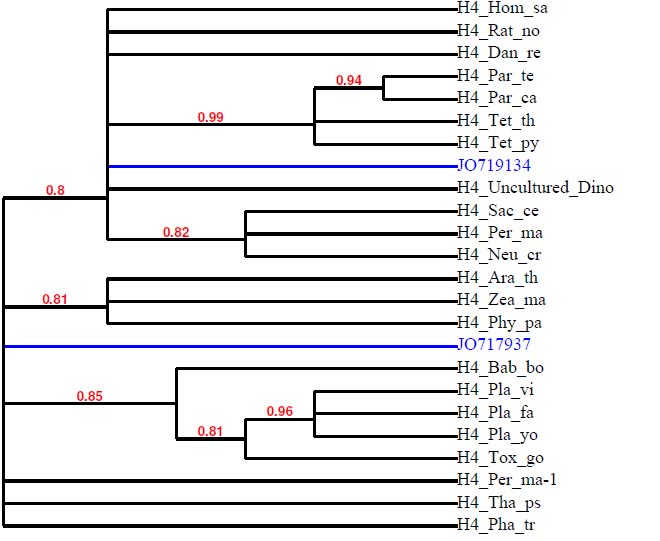

Supplement: Figure S3 — Cladogram of histone H4. The cladogram of histone sequences shows representatives from mammals, plants, fungus and members of the superphylum Alveolata. The representative sequences were obtained from Pubmed database and bear the first three letters from genus followed by two letters from species. The values in red at each node indicate the respective Bootstrap support value. Lingulodinium sequences are coloured in blue. (JPG) [file pone.0034340.s003.jpg]

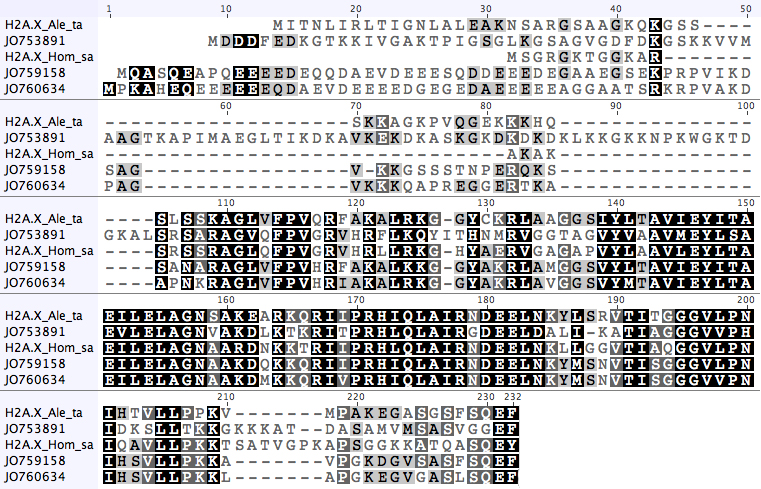

Supplement: Figure S5 — Alignment of H2A sequences. Multiple sequence alignment of histone H2A from yeast, human and Lingulodinium is shown. (JPG) [file pone.0034340.s005.jpg]

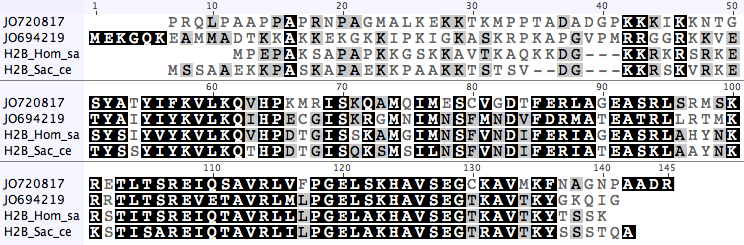

Supplement: Figure S6 — Alignment of H2B sequences. Multiple sequence alignment of histone H2B from yeast, human and Lingulodinium is shown. (JPG) [file pone.0034340.s006.jpg]

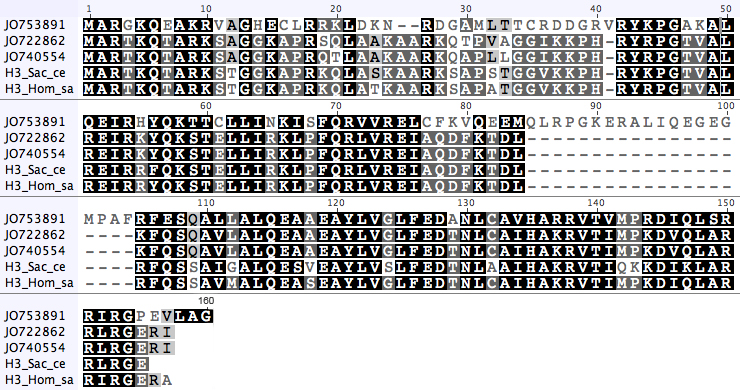

Supplement: Figure S7 — Alignment of H3 sequences. Multiple sequence alignment of histone H3 from yeast, human and Lingulodinium is shown. (JPG) [file pone.0034340.s007.jpg]

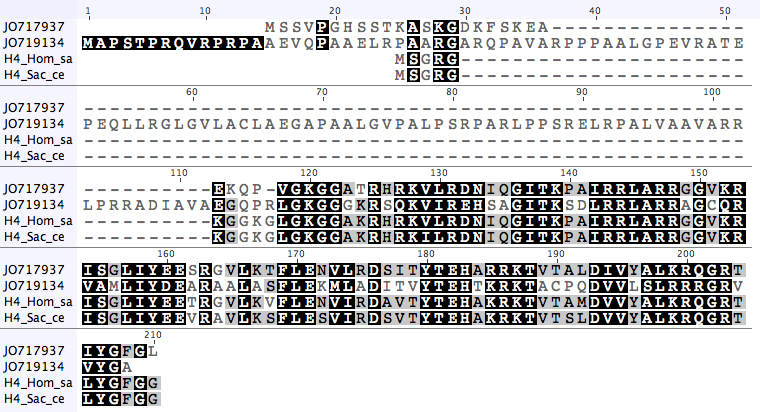

Supplement: Figure S8 — Alignment of H4 sequences. Multiple sequence alignment of histone H4 from yeast, human and Lingulodinium is shown. (JPG) [file pone.0034340.s008.jpg]
