# Supplementary material for: A Full Suite of Histone and Histone Modifying Genes Are Transcribed in the Dinoflagellate Lingulodinium
Source: PLoS One. 2012 Apr 4;7(4):e34340. doi: 10.1371/journal.pone.0034340 (PMC3319573; doi:10.1371/journal.pone.0034340)
Supplement: Figure S4 — Histone H2B protein is not detected in Lingulodinium (TIFF). Western blotting with H2B antibody is shown here. The amount of protein (in micrograms) per lane is written above each lane. (PDF) [file pone.0034340.s004.pdf]

```
H4_27324_L        mapstprqvrprpaaevqPaaelrpAarGarqpavArppPaalgpevratepeqllrglg
H4_25974_1        --------------MSsvPghsstKAsKGDKfSKEAEKQP--------------------
H4_25974_2        --------------MSsvPggsstKAsKGDKfSKEAEKQP--------------------
H4_Hom_sa         -------------------msGrgKgGKG-------------------------------
H4_Sac_ce         -------------------msGrgKgGKG-------------------------------

H4_27324_L        vlaclaegapaalgvpalpsrparlppsrelrpalvaavarrlprradiavaegqprLGK
H4_25974_1        ---------------------------------------------------------vGK
H4_25974_2        ---------------------------------------------------------vGK
H4_Hom_sa         ---------------------------------------------------------LGK
H4_Sac_ce         ---------------------------------------------------------LGK

H4_27324_L        GGgKRsqKViRehsaGITKsdlRRLARRaGcqRvamLIYdEARaaLaSFLEkmLaDitvY
H4_25974_1        GGAtRHRKVLRDNIQGITKPAIRRLARRGGVKRISGLIYEESRGVLKTFLENVLRDSITY
H4_25974_2        GGAtRHRKVLRDNIQGITKPAIRRLARRGGVKRISGLIYEESRGVLKTFLENVLRDSITY
H4_Hom_sa         GGAKRHRKVLRDNIQGITKPAIRRLARRGGVKRISGLIYEETRGVLKvFLENViRDAVTY
H4_Sac_ce         GGAKRHRKiLRDNIQGITKPAIRRLARRGGVKRISGLIYEEvRaVLKSFLEsViRDSVTY

H4_27324_L        TEHtKRKTacpqDVVlsLrRrGRvVYGa--
H4_25974_1        TEHArRKTVTALDiVYALKRQGRTIYGFGl
H4_25974_2        TEHArRKTVTALDiVYALKRQGRTIYGFGl
H4_Hom_sa         TEHAKRKTVTAMDVVYALKRQGRTlYGFGg
H4_Sac_ce         TEHAKRKTVTsLDVVYALKRQGRTlYGFGg
```
