# Supplementary material for: A Full Suite of Histone and Histone Modifying Genes Are Transcribed in the Dinoflagellate Lingulodinium
Source: PLoS One. 2012 Apr 4;7(4):e34340. doi: 10.1371/journal.pone.0034340 (PMC3319573; doi:10.1371/journal.pone.0034340)
Supplement: Table S1 — LC-MS/MS identification of acid soluble proteins from Lingulodinium extracts fractionated on SDS PAGE. (DOCX) [file pone.0034340.s009.docx]

**Supplemental Table 1. LC-MS/MS identification of acid soluble proteins from *Lingulodinium* extracts fractionated on SDS PAGE**

| **Type of Protein** | **No. of Proteins (≥2 peptides /protein)** | **Confidence (e-value)** | **Species Hit** |
| --- | --- | --- | --- |
| ***Lingulodinium polyedrum*** | | | |
| **Unknown/Hypothetical** | **71** | **-** | **-** |
| **PS II cytochrome c550** | **1** | **5.5e-30** | ***Kryptoperidinium foliaceum*** |
| **Ribosomal protein L18** | **1** | **6.7e-20** | ***Ostreococcus lucimarinus*** |
| **Calmodoulin** | **1** | **9.4e-27** | ***Pfiesteria piscicida*** |
| **HSP70** | **1** | **1.4e-11** | ***Prorocentrum minimum*** |
| **HMG Box 2** | **1** | **5.7e-11** | ***Xenopus (Silurana) tropicalis*** |
| **Oxygen evolving enhancer** | **1** | **2.9e-60** | ***Heterocapsa triquetra*** |
| **40S ribosomal protein S9** | **1** | **9.6e-40** | ***Plasmodium knowlesi*** |
| **Chromosome segregation protein** | **1** | **6e-05** | ***Haloarcula marismortui*** |
| **60S ribosomal protein L7** | **1** | **1.6e-24** | ***Perkinsus marinus*** |
| **Ribosomal protein L23a** | **1** | **2.6e-38** | ***Rhodomonas sp.*** |
| **Adenosylhomocysteinase** | **1** | **7.2e-67** | ***Amphidinium carterae*** |
| **Chloroplast PSII subunit III** | **1** | **2.2e-52** | ***Heterocapsa triquetra*** |
| **Chloroplast phosphoglycerate kinase** | **1** | **2.8e-63** | ***Heterocapsa triquetra*** |
| **Glyceraldehyde-3-phosphate dehydrogenase** | **2** | **1.15e-72; 2e-63** | ***Gonyaulax polyedra*** |
| **Elongation factor 1α-like** | **1** | **2.5e-32** | ***Heterocapsa triquetra*** |
| **40S ribosomal protein S15** | **1** | **5.8e-32** | ***Perkinsus marinus*** |
| **40S ribosomal protein S3** | **1** | **4.3e-85** | ***Ricinus communis*** |
| **Light harvesting protein** | **1** | **1e-68** | ***Symbiodinium sp.*** |
| **Glutaredoxin related protein** | **1** | **3e-37** | ***Oxyrrhis marina*** |
| **D3 phosphoglycerate dehydrogenase** | **1** | **1.6e-32** | ***Perkinsus marinus*** |
| **ADP ribosylation factor** | **2** | **5.3e-62; 3.1e-61** | ***Pfiesteria piscicida*** |

An acid extracted protein sample was electrophoresed on SDSPAGE and the bands corresponding in size to yeast histones excised and subjected to trypsin digestion for identification of peptide sequence by LC-MS/MS. A summary of all proteins identified by a least two peptides is shown.
